# A Review of the Quiz, as a New Dimension in Medical Education

**DOI:** 10.7759/cureus.18854

**Published:** 2021-10-18

**Authors:** Chetna Dengri, Akshay Gill, Jayesh Chopra, Chestha Dengri, Thoyaja Koritala, Anwar Khedr, Aishwarya Reddy Korsapati, Ramesh Adhikari, Shikha Jain, Simon Zec, Mool Chand, Rahul Kashyap, Vishwanath Pattan, Syed Anjum Khan, Nitesh K Jain

**Affiliations:** 1 Medicine, University College of Medical Sciences, Delhi, IND; 2 Neurology Department, Sir Gangaram Hospital, Delhi, IND; 3 Internal Medicine, University College of Medical Sciences, Delhi, IND; 4 Medicine, Maulana Azad Institute of Dental Sciences, Delhi, IND; 5 Department of Hospital Medicine, Mayo Clinic Health System, Mankato, USA; 6 Department of Critical Care Medicine, Mayo Clinic Health System, Mankato, USA; 7 Internal Medicine, Tanta University Faculty of Medicine, Tanta, EGY; 8 Department of Internal Medicine, University of Buckingham Medical School, London, GBR; 9 Hospital Medicine, Franciscan Health, Lafayette, USA; 10 Geriatrics, Brown University, Providence, USA; 11 Internal Medicine, MVJ Medical College, Bengaluru, IND; 12 Department of Critical Care Medicine, Mayo Clinic, Rochester, USA; 13 Division of Critical Care Medicine, Mayo Clinic, Rochester, USA; 14 Endocrinology, Wyoming Medical Center, Casper, USA

**Keywords:** medical quiz, teaching-learning tools, higher education medical training, customized medical education, didactic lecture, student-centric learning, medical jeopardy, medical student performance evaluation, medical student training

## Abstract

Over the years, medical education delivery has seen a change from teacher-centric to student-centric teaching-learning methods. Educators are constantly looking to develop interactive and innovative teaching-learning tools. One such supplementary tool is the use of the quiz for medical education. The Quiz has been used traditionally as a feedback assessment tool, but lately, it has found its way into the medical curriculum, mostly informally. The few available documented studies on the Quiz as a teaching and learning tool illustrate its acceptance and impact on the stakeholders. It could be one of the solutions to the endless search for a student-centric and engaging tool to deliver the medical curriculum. Commonly, the format for medical quiz is either on a case-based or image-based approach. Such an approach helps bridge the gap between traditional classroom teaching and clinical application. The Quiz is a readily acceptable tool that complements didactic lectures and improves students' learning and comprehension. Being an interactive student-centric tool, it enhances active student participation and encourages regular feedback mechanisms. It promotes healthy competition and peer-assisted learning by encouraging active discussion among students, hence improving student performance in standard examination techniques, along with teacher satisfaction. This literature review aims to enumerate the various formats of the Quiz, their role in improving the understanding and retention of knowledge among the students and assess their acceptability among the stakeholders.

## Introduction and background

Instructor-driven didactic lectures have been the most prevalent teaching mode in medical education across the world over the last few decades. It has been increasingly recognized that didactic lectures are a poor method for exchanging information [1]. Students find lectures to be generally an inefficient mode of learning [2]. However, the teacher-centered, passive didactic lectures continue to be the most common mode of medical teaching, comprising the age-old one-way flow of information. This often traps disinterested students in a non-participatory relationship, leading to disinterest and inefficient use of time and resources. So, there is a need for a structural framework in medical education to build a learner or student-centric, context-oriented system to impart knowledge and measure outcomes [3].

Several alternative teaching-learning tools, such as flipped classroom teaching, small group learning, community placement, personal reflection, tutorials, demonstrations, etc., have been proposed instead of lectures around the world. However, none of them could gain student popularity [4], or in other words, make a positive impact on student population. Educators have struggled to develop student-centered and problem-oriented learning tools to stimulate active student participation [5].

The "Quiz" as a word appeared around 1780s, as student colloquialism, without any proper definition. But over time, it established itself with other well-known terminologies of evaluation such as "Test" and "Examination." It is now defined as a "Test of knowledge," traditionally Quiz has been associated as a testing entity for trivial pursuit, for assessment and feedback purposes [6]. In the present narrative review, we evaluated the available literature on the role of Quiz as a tool of teaching and learning in medical education. It assesses the various formats of the Quiz, their role in improving the understanding and retention of information among the students, and their acceptability among the stakeholders.

## Review

Methodology and results:

We designed this study to be a narrative review, with a comprehensive analysis of the articles based on the studies in which various formats of Quiz were employed as a teaching and learning tool. Subsequent observations were made of the impact on participants (or students), their feedback, and acceptability towards these methods. Our primary intention was to screen for articles that employed the Quiz for teaching and learning purposes and had valuable inferences from the study based on factors like knowledge testing, knowledge retention, competitiveness, amount of participation, and being student centric. The purpose of the search of these factors in the articles was to see the difference that the Quiz as a teaching-learning tool had compared to conventional methods.

**Figure 1:**
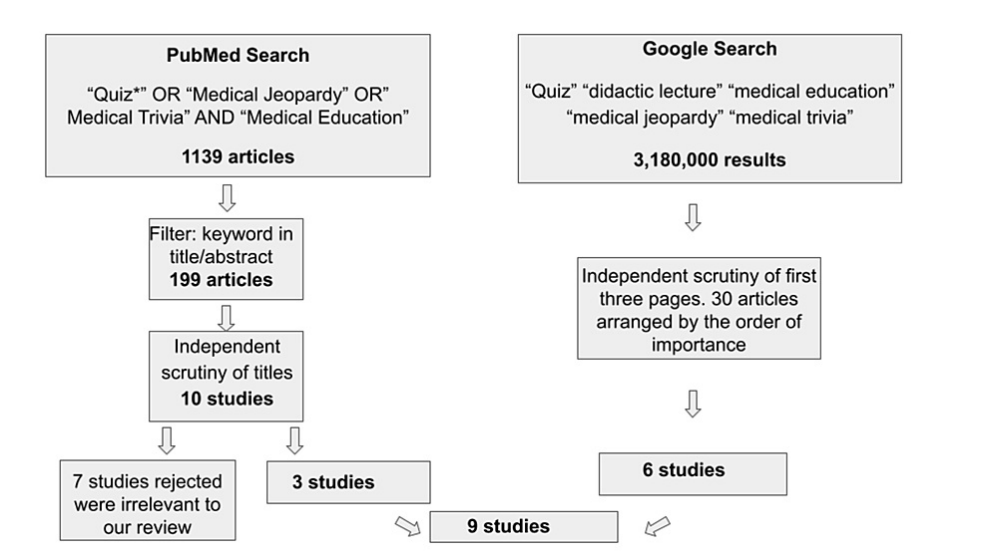
Flow of Literature search using PubMed and Google in August 2021.

We carried out the literature search in August 2021 on the platforms: PubMed and Google search. On PubMed database, using the keywords "Quiz" or "Medical Jeopardy" or "Medical Trivia" and "Medical Education" a search was done, which revealed 1139 citations. On limiting the search to the keywords appearing in either the title or abstract, 199 articles were identified. Independent scrutiny of the title and abstract of these articles helped us identify five articles relevant to our aim and objective. Further, a Google search with the keywords "Quiz," "Didactic lecture," "Medical Trivia," "Medical Jeopardy," and "Medical Education" revealed 3,180,000 web pages arranged in the order of importance as estimated by PageRank. The title of the web pages appearing in the first 30 web pages helped us add another six relevant studies. We excluded one study as it followed a case-based approach to teach; however, it did not follow a competitive quizzing atmosphere. Another study was excluded as its studies design used the Quiz in both arms, i.e., paper Quiz in the control arm, while web-based Quiz in the intervention arm. The flow of the search is represented in Figure 1. We reviewed the identified articles and extracted information with a focus on the format of the Quiz, the impact of the Quiz on student performance, and the acceptability of the Quiz as a teaching-learning tool among the students and teachers.

 

In summary, after scrutinizing 199 published articles on PubMed and 30 articles on Google search, we identified 11 relevant articles, which have evaluated the performance of the Quiz as a teaching and learning tool in medical education that was related to our study with a focus on format, impact, and acceptability. The format for medical quizzing has been on a case-based/image-based approach, bridging the gap between classroom teaching and clinical application. Table 1 presents the key findings from the studies included in our review. 

**Table 1:** Summary of studies that used the Quiz as a teaching-learning tool * indicates score out of 100

Author, year, and journal	Participants	Objective(s)	Methodology	Results
Talsania N. et al. l, 2015. International Journal of Scientific Study, India. [7]	121 MBBS students in control arm. 121 MBBS students in intervention arm.	To ascertain the effectiveness of quizzes as an interactive teaching technique. To implement various quizzing activities in lectures and evaluate their impact on learning.	Cross-sectional comparative and interventional study	Attendance rate: Control (C): 68%. Intervention (I): 75%. Pre-test score*: C: 33% scored between 41-60. I: 52% scored less than 40, 36% students scored between 41-60. Post-test score*: C: none scored above 80, 48% scored between 41-60. I: 46% scored between 81-100, 35% scored below 40. Participant feedback: the student response was overall positive. Respondents found the quiz engaging, innovative, interactive, liked the scope for participation, found the competition healthy, and an exemplary method for learning. Suggestions were to have quizzes for other topics.
Devi K. et al., 2014. National Journal of Community Medicine, India. [8]	151 MBBS students.	To increase awareness and interest in the topic. To increase knowledge and promote the application of knowledge students. To enhance student participation by using Quiz as a method to teach.	Descriptive study	Attendance rate: 92%. Average pre-test score: 33.3%. Average post-test score: 98.6%. Participant response: participants suggested having quizzes for other subjects, found them interesting, innovative, interactive, the scope for participation, promote healthy competition, a suitable learning method.
Lauw. M. N et aI, 2011. The Journal of Medicine, Netherlands [9].	452 doctors from various sub-specialties.	Using clinical images and tests to enhance memorization of facts and information in medical education by giving a weekly medical quiz.	Weekly clinical cases in quiz emailed to the registered participants for two years.	Average response: 33/452 people per case [7.3% (4.9-9.7)]. Cases with high response rates were associated with more correct answers than cases with low response rates. Specialists were more likely to respond to cases from their sub-specialty. Most answers were submitted on the same day.
Khan S et al., 2017. Indian Journal of Orthodontics and Dentofacial Research. [10]	24 BDS students.	To evaluate quiz as a teaching-learning technique. To assist students in the clinical application of study knowledge.	Comparative and interventional study.	Attendance rate: 83%. Average pre-test score*: 40+25. Average post-test score*: 82+7. Participant feedback: students were found to be more attentive, showed increased interest and liked the interactive techniques.
Khan M et al., 2011. Saudi Medical Journal. [11]	41 fifth-year medical students in the control arm. 41 fifth-year medical students in intervention arm.	To compare students' performance, satisfaction, and knowledge retention between a jeopardy game format and a didactic lecture format.	Randomized control trials.	Both groups showed significant improvement in their knowledge on the post-test compared with the pre-test scores. The post-test II conducted after two months showed that knowledge retention was significantly better in the jeopardy game format. The satisfaction survey showed that the jeopardy game format was more enjoyable.
Aljezawi et al., 2015. British Journal of Nursing. [12]	32 nursing students in the control arm. 34 nursing students in the intervention group.	To compare students’ performance, satisfaction, and knowledge retention between a jeopardy-style game format and a didactic lecture format.	Parallel group randomized controlled trials.	Pre-test results showed no significant difference between the two groups. In the immediate post-test and the retention test, the students in the jeopardy style quiz group scored significantly better than those in the lecture group. Participant feedback: a satisfaction questionnaire showed that the jeopardy-style game format was well-liked and accepted by students as a more satisfying teaching method.
Friedrich S. et al., 2019. Journal of Dental Problems and Solutions. [13]	25 first-year medical students.	To investigate the learning success in first clinical semester dentistry students through game-based learning in the "Jeopardy"-setting. To determine the satisfaction and learning behavior of the students.	Randomized experimental pilot study.	Comparison of pre-test and post-test scores demonstrated an average score increase by 2.08 points (P), score of the active player group improved by 0.31P, while the passive listener group improved by 4P. This highlights a positive impact of game-based learning on passive listeners as well. Participant feedback: all participants assessed the session (7.64+1.8 on a scale of 1-10). Session stimulated self-study (4.2+0.9 on a scale of 1-5). A majority indicated that they would recommend a game-based setting to other students (4.0+0.9 on a scale of 1-5).
Ellis G et al., 2020. MedEd Portal, The AAMC Journal of Teaching, and Learning Process. [14]	26 family medicine residents.	To assess the residents' enjoyment of and learning from the activity using a Likert scale-based feedback form tool.	Descriptive study.	All the residents agreed or strongly agreed that the session was an enjoyable opportunity for learning and something they would look forward to in the future. All the residents also agreed that the information presented applied to clinical practice.
Kunzler E et al., 2016. Dermatology Online Journal. [15]	143 medical students.	To explore if creating healthy competition with small incentives can affect student participation through a weekly online quiz for 10-weeks.	Cross-sectional study.	An average of 23.8% of the medical student participated in the quiz. The least participation was recorded in the first week (5.6%); however, participation increased in the subsequent weeks ranging from 21.7% to 30.1%. Maximum participation was reported in second week (30.1%). Optional quiz sessions with small incentives positively impacted motivational competition and interaction with faculty, while serving as a source of education.

Discussion:

The Quiz is a unique and interactive method of teaching and learning and follows a question-answer format, thus increasing active participation and healthy competition. The Quiz can be conducted using various question types, i.e., multiple-choice, true/false, short answers, multiple responses, fill in the blanks, matching, sequence, etc. The use of these interactive formats provides immediate feedback to the students and helps direct the conversation among students. The use of Quiz for teaching-learning provides two advantages. It helps assess the current knowledge base and then helps build on it by active student interaction. Student-organized quizzing events promote leadership qualities, innovation, teamwork, time management, and organizational skills among medical students [16]. A significant increase in the post-test score, which indicates a better understanding of the topic, has been almost universally reported, along with encouraging student feedback in the studies reviewed in this article.

In the following section, we present the different formats of quizzes that could be used, the impact, and the acceptability of Quizzing as a teaching-learning tool. 

Format:

A didactic lecture can be conducted only in one form, i.e., unidirectional flow of information, making it less interactive. Especially during the pandemic, when virtual learning is the new norm, it is essential to develop and use interactive teaching tools that can capture the attention of remotely located students surrounded by various distractions at home. The Quiz has the flexibility and freedom of being conducted online or offline and in various formats, such as written or oral; image-based, case-based, rapid-fire questions, crosswords, or even in a game-like pattern with enormous innovation potential [17].

Talsania et al. in India conducted a study having a total of 242 participants; half of the students were taught by conventional didactic lecture, while the other half by the quiz method. The Quiz consisted of the following rounds: multiple choice questions (MCQs), visual-based, short answer, and rapid-fire. On receiving feedback, many of the students liked the rapid-fire round (48%), closely followed by visual format (28%), and MCQ format (19%) [7]. Feedback from students who participated in the descriptive study by Devi et al. revealed the preference of various formats in the following order: rapid-fire, visual format, MCQ format, and only six students preferred short answer format [8]. Lauw et al. conducted a weekly online quiz via email for two years by sending clinical cases and images to 452 registered participants consisting of residents and staff members from various subspecialties of Internal medicine at an academic medical center located in the Netherlands. Of the 452 registered members, only an average of 33 (7.3%) responded per case; this low response rate was attributed to time constraints and the fear of failure among peers. However, the cases with high response rates were associated with a higher percentage of correct answers (63.8%) than cases with low response rates (percentage of the correct answers was 45%). Also, residents were more likely to answer when the case was from their sub-specialty [9].

Impact:

Various studies show a significant improvement in understanding and retention of knowledge after teaching sessions were conducted using quizzes. 

Devi et al. conducted a descriptive study in 2014 with 151 medical students, intending to increase awareness, interest, and application of knowledge by using quizzing as a teaching-learning tool. In this study, the pre-test score of most students (73%) was between 0%-40%, and only 3% of students scored in the range of 61%-80%. However, the post-test score of the same students after the quizzing session showed a substantial improvement, with 93% of students scoring above 81% [8]. Khan S. et al. conducted a lecture quiz series on 24 dental students with the format consisting of two-hour didactic lectures, followed two weeks later by a quiz session on the same topic. The pre-test score was 40+25 out of 100, while the post-test score was 82+7 out of 100, which substantially improved. In addition, the narrowed standard deviation suggested uniform distribution of information among the students [10]. Khan M.N. et al. conducted a parallel-group, randomized controlled trial on 82 fifth-year medical students. Forty-one students in the control arm were taught via lecture format, while 41 students in the intervention group participated in the “Jeopardy” style quiz on a topic. It was found that there was a significant improvement in the immediate post-test score, in comparison to the pre-test score between the control and intervention group, favoring the intervention group. Additionally, the intervention group reported significantly better knowledge retention than the control group in a second post-test evaluation conducted two months after the session [11]. A similar study by Aljezawi et al. on 66 nursing students found no significant difference in pre-test scores of control and interventional arm; however, post-test scores and retention test scores were significantly better in the intervention arm [12]. A randomized experimental pilot on 25 first-semester dental students was conducted by Friedrich S. et al. It was found that average scores of pre-tests and post-tests showed an improvement of 2.08 points. This study highlighted that use of a game-based learning approach impacted active participants in the session and the passive listeners. Analysis of the post-test results revealed that the active player group improved by 0.31 points, while the passive listener group improved by four points [13].

Acceptability:

One of the critical factors to assess the acceptability of the Quiz was to take into consideration the attendance rate after prior notification. 

A cross-sectional comparative and interventional study conducted in 2015 by Talsania N et al. showed a significant difference between the attendance rate of the control arm (68%) and the intervention arm (75%) after two-week prior notifications [7]. Similarly, a near-perfect attendance rate (92%) was seen in the descriptive study by Devi et al. [8]. Khan S. et al. conducted a cross-sectional, comparative, and interventional study in 2017, and feedback by the participating students was encouraging, as many stated that their active participation helped shape the lecture in a format best suited for their requirements. Also, the students found the learning session interactive and friendly and paid more attention to the discussion than a didactic lecture. The faculty involved developed the Quiz in an organized, better coordinated, and well-sequenced manner, which helped the participants broaden the spectrum of their knowledge [10]. The feedback from the participating medical students in the studies conducted by Devi K. and Talsania N. et al. was also encouraging. Students from both studies appreciated quizzing as an effective, friendly, and interactive learning mode, ensuring healthy competition and active learning. The only suggestion was to organize quizzing more frequently in the institution [7,8]. Ellis G. et al. implemented a Jeopardy-style game during a one-hour didactic session for 26 family medicine residents. Feedback and learning for the activity were assessed using a session-specific evaluation tool. It was found that the residents highly accepted the Jeopardy game session. In addition, all participants found the session as ‘an enjoyable opportunity for learning,’ and they agreed that the information presented applied well to clinical practice [14]. In a study by Kunzler E. et al., 143 medical students were given an option to participate in a quiz. The objective was to impart education by promoting healthy competition with small incentives in a 10-week online quiz activity. Although, on average, only 23.8% students participated in this quiz activity, there was a gradual increase in participation, with a positive impact in learning in this cross-sectional study [15].

Our review has certain strengths and limitations. In terms of strength, it is an exhaustive and contemporary review of published literature in health sciences that explores the potential of Quiz as a tool for providing education that is student-centric and engaging. Indeed, as discussed, there appears to be convincing evidence that this is an effective strategy. The limitations of our review are that different studies have different methodologies, they have a few participants and lack long-term longitudinal follow up. Future studies should focus on these limitations while designing and conducting studies on this subject.

## Conclusions

It is evident that students find the Quiz-based learning more engaging, interactive, and it increases student participation, curiosity, and eagerness to learn. The format of the Quiz can be changed to meet the specific goals based on the target audience and the objective that the teacher wants to meet. The Quiz based learning can be more effective by keeping it dynamic and flexible, which means the format, content, and level of difficulty should be titrated to the needs, strengths, and weakness of the target audience or students.

## Appendices

Authors' Contributions: Conceptualization: Chetna Dengri and Nitesh Jain; Methodology: Akshay Gill, Jayesh Chopra; Validation: Nitesh Jain, Thoyaja Koritala; Investigation: Thoyaja Koritala; Data Curation: Anwar Khedr, Aishwarya Reddy Korsapati, Ramesh Adhikari, Shikha Jain; Writing (Original Draft): Chetna Dengri, Akshay Gill, Jayesh Chopra, Chestha Dengri, Aishwarya Reddy Korsapati, Rahul Kashyap; Writing (Review & Editing): Thoyaja Koritala, Ramesh Adhikari, Shikha Jain, Simon Zec, Mool Chand, Vishwanath Pattan, Syed Anjum Khan, Nitesh K. Jain; Visualization: Chetna Dengri and Nitesh Jain; Supervision: Syed Anjum Khan and Nitesh Jain; Project administration: Nitesh Jain, Chetna Dengri; Funding acquisition: NA
